# Coronary health index based on immunoglobulin light chains to assess coronary heart disease risk with machine learning: a diagnostic trial

**DOI:** 10.1186/s12967-024-06043-4

**Published:** 2025-01-06

**Authors:** Wenbo Ren, Zichen Zhang, Yifei Wang, Jiangyuan Wang, Li Li, Lin Shi, Taiyu Zhai, Jing Huang

**Affiliations:** 1 Department of Clinical Laboratory, The First Hospital of Jilin University, Changchun, 130000 China; 2 College of Medical Technology, Beihua University, Jilin, 132000 China; 3 Department of Clinical Laboratory, Lequn Branch, The First Hospital of Jilin University, Changchun, Jilin 130000 China

**Keywords:** Coronary health index, Coronary heart disease, Machine learning models, Immunoglobulin light chains, High-risk

## Abstract

**Background:**

Recent studies suggest a connection between immunoglobulin light chains (IgLCs) and coronary heart disease (CHD). However, current diagnostic methods using peripheral blood IgLCs levels or subtype ratios show limited accuracy for CHD, lacking comprehensive assessment and posing challenges in early detection and precise disease severity evaluation. We aim to develop and validate a Coronary Health Index (CHI) incorporating total IgLCs levels and their distribution. Additionally, we aim to evaluate its effectiveness by integrating patient data and using machine learning models through diagnostic trial.

**Methods:**

The CHI was developed and combined with other clinical data. Nine machine learning models were screened to identify optimal diagnostic performance, with the XGBoost model emerging as the top performer. Performance was assessed based on accuracy, sensitivity, and its ability to identify severe CHD cases characterized by complex lesions (SYNTAX score > 33).

**Results:**

The XGBoost model demonstrated high accuracy and sensitivity in diagnosing CHD, with an area under the curve (AUC) of 0.927. It also accurately identified patients with severe CHD, achieving an AUC of 0.991. An online web tool was introduced for broader external validation, confirming the model’s effectiveness.

**Conclusions:**

Combining the CHI with the XGBoost model offers significant advantages in diagnosing CHD and assessing disease severity. This approach can guide clinical interventions and improve large-scale CHD screening.

**Supplementary Information:**

The online version contains supplementary material available at 10.1186/s12967-024-06043-4.

## Background

Cardiovascular disease, particularly coronary heart disease (CHD), poses a formidable global challenge, both in treatment and prevention [1]. The intricate treatments, coupled with costly interventions and often asymptomatic early stages, make managing and mitigating its incidence exceedingly difficult, posing a significant threat to global health [2]. According to estimates, CHD claimed the lives of 9.14 million people globally in 2019, while 197 million individuals were living with the condition. This burden is escalating rapidly in low- and middle-income countries such as China and Southeast Asia [3]. The alarming incidence and mortality rates underscore the urgent need for efficient diagnostic and therapeutic strategies to manage CHD effectively. Furthermore, CHD encompasses a wide spectrum of myocardial ischemia conditions resulting from coronary artery atherosclerosis, ranging from minor myocardial insufficiency (angina pectoris) to severe myocardial necrosis (myocardial infarction) [4]. The majority of clinicians have profoundly acknowledged the pivotal role of precision medicine in enhancing patient treatment outcomes and quality of life. However, we face a novel challenge: how to promptly and conveniently differentiate CHD patients based on varying degrees of severity, as this differentiation directly impacts the selection of the most suitable treatment modalities [5]. Nevertheless, we still have significant deficiencies in this area at present.

Although notable advancements have been made in the diagnosis and treatment of CHD over the past several decades, numerous challenges persist. CHD diagnosis has traditionally relied on imaging techniques, and although many are noninvasive, such as echocardiography and computed tomography, can clearly visualize the structure and function of the heart without harming the patient, effectively identifying coronary artery stenosis and plaque formation. Nevertheless, these techniques are not flawless, as the presence of false positives and false negatives may lead to diagnostic errors. Others are invasive and carry high risks, like coronary angiography and intravascular ultrasound offer more precise diagnostic information. However, their procedures are accompanied by risks, including vascular puncture, bleeding, infection, or allergic reactions that may arise from the use of contrast agents, causing additional discomfort and burden to patients; additionally, all techniques are costly and equipment-dependent, limiting access [6, 7].

Other studies have demonstrated the successful application of biochemical markers, such as troponin and brain natriuretic peptide (BNP), in diagnosing CHD. However, despite their desirable diagnostic performance, these biochemical markers currently in use in clinical practice, such as troponin and BNP, also have some limitations. Troponin, for example, is primarily employed for diagnosing acute coronary syndrome but is insufficient for long-term prognosis prediction or overall risk assessment in CHD. Similarly, the specificity of BNP is a crucial biomarker in the assessment of heart failure, where elevated levels of BNP often signify the presence of left ventricular dysfunction and increased cardiac stress, however, it is limited because its serum concentration is influenced by various noncardiac factors [8, 9]. Consequently, researchers are now aiming to uncover novel biomarkers that can be rapidly and noninvasively measured and are highly specific and sensitive; such biomarkers would not only increase diagnostic precision but also potentially reduce diagnostic costs, thus benefiting a larger patient population.

Extensive research has confirmed the relationships between atherosclerotic diseases, including CHD, and the immune system, highlighting the crucial roles played by different components of the immune system in the initiation, development, and progression of atherosclerosis [10]. Developments in the field of immunology have garnered immunoglobulin light chains (IgLCs) significant attention from researchers as a biomarker of great potential. Alterations in the levels of total immunoglobulin light chains (TLCs) and free immunoglobulin light chains (FLCs) have been used as key indicators for monitoring disease activity in conditions such as multiple myeloma and certain autoimmune disorders [11, 12]. IgLCs are crucial components of the immunoglobulins produced by B cells and play indispensable roles in the immune system. Each immunoglobulin molecule is composed of exactly two heavy chains and two light chains, the latter of which exist in two subtypes, κ and λ [13]. In healthy individuals, IgLCs exist in a stable proportion, the κ:λ light chain ratio is approximately 2:1 [14].

Studies have revealed an unexpected association between IgLCs and cardiovascular events, potentially indicating their role as important biomarkers for cardiovascular disease (CVD) [15, 16]. Patients with heart failure, for example, have presented with abnormal IgLCs levels that are highly correlated with disease severity and the probability of adverse clinical outcomes [17]. Another study found that high levels of serum free κ light chains are independently associated with the presence of CVD, potentially indicating their suitability as biomarkers for early disease detection [18]. Similarly, there seems to be a potential connection between IgLCs and CHD. Existing studies have confirmed the correlation between variations in IgLCs levels and the occurrence, progression, and prognosis of CHD. As early as 2015, a study already found that, in patients with ST-elevation myocardial infarction (STEMI), the levels of FLCs could predict the future necessity for percutaneous coronary intervention (PCI) [19]. Another recent study has shown that, in CHD, patients with elevated κ/λ ratios had an improved left ventricular ejection fraction. These serum FLCs have also been suggested to play pivotal roles in the pathogenic mechanisms underlying the disease [20]. However, despite the correlation between IgLCs and CHD, the application of IgLCs in diagnosing CHD still faces challenges. The accuracy of predicting risk using IgLCs levels or subtype ratios alone is insufficient, primarily due to significant individual differences influenced by genetics, environment, lifestyle, and other factors. Additionally, the pathogenesis of CHD is complex, encompassing inflammation, lipid metabolism, endothelial dysfunction, platelet aggregation, and other processes. Therefore, for predicting and diagnosing CHD, a deeper understanding of the interactions between IgLCs and other biomarkers as well as clinical indicators is required, and more comprehensive and precise diagnostic strategies need to be explored.

Machine learning (ML) models offer a novel solution for enhancing diagnostic accuracy by analyzing large datasets to precisely identify subtle differences among biomarkers, thereby strengthening the discriminatory power of diagnosis [21]. This study aims to leverage algorithmic advantages to explore and optimize the utilization of IgLCs in distinguishing CHD patients. Although IgLCs hold immense potential as biomarkers for cardiovascular diseases, there remains a need for further research on how to optimally utilize them to differentiate CHD patients from high-risk individuals and for initial classification of CHD patients. Additionally, the application of ML models in improving the diagnostic accuracy and predictive capability based on IgLCs methods is yet to be fully explored.

To address this gap, in our study, we have constructed a novel assessment tool named the “Coronary Health Index” (CHI), grounded on the distinctive variations of the two light chain subtypes of IgLCs within the human body. Furthermore, we intend to integrate the CHI with multifaceted clinical data and employ machine learning to develop a predictive model. Our central objective is to utilize this model to comprehensively and systematically evaluate the efficacy of IgLCs in diagnosing CHD, while also delving into their potential value in CHD screening, early detection, as well as stratifying disease severity and progression. In this study, our model has preliminarily demonstrated its potential role in stratifying the severity or progression of CHD. We anticipate that this research will deepen our scientific understanding of how IgLCs influence the pathogenesis of CHD and provide insights and inspiration for precision management and optimized treatment strategies of cardiovascular diseases.

## Methods

### MethodsStudy subject inclusion and exclusion criteria

In this study, gender was not considered as a biological variable. This study was approved by the institutional review board and ethics committee of the First Hospital, Jilin University (2024 − 729) and was conducted according to the ethical standards established in the Declaration of Helsinki. Informed consent was obtained from all participants. We aim to investigate the diagnostic effectiveness of IgLCs by retrospectively recruiting patients suspected of CHD who were admitted to the First Hospital of Jilin University from January 2024 to April 2024.

The inclusion criteria for the CHD patient group were rigorously defined to ensure the validity and reliability of the study results. Specifically, the inclusion criteria for the CHD patient group included the presentation of relevant symptoms (such as chest pain, n, shortness of breath, fatigue, or palpitations), age below 80 years, a suitable physical condition for undergoing coronary angiography, and first-time CHD with no previous treatment history. Patients who had taken anticoagulant medications, such as aspirin, in the three months prior to the study, those with a poor overall condition, those who were unsuitable for surgical treatment, or those with other contraindications for blood sampling were excluded. All suspected CHD patients after enrollment need to undergo coronary angiography, and volunteers who do not obtain this data will also be excluded from the study cohort. For the volunteers finally included in the CHD group, we will further categorize them into three subgroups based on their SYNTAX scores, including mild lesions (SYNTAX score = 1–22), moderate lesions (SYNTAX score = 22.5–32), and severe lesions (SYNTAX score = 32.5–49.5).

For the control group of this study, we selected patients who were admitted with suspected CHD due to other similar symptoms but were confirmed to have no abnormalities in their coronary arteries after undergoing coronary angiography. These patients may exhibit similar symptoms to those in the CHD patient group, but they were excluded from a CHD diagnosis based on the absence of coronary stenosis or obstruction, as confirmed by rigorous coronary angiography combined with other clinical manifestations. The baseline information and other examination results of the volunteers in the two groups who were ultimately enrolled underwent desensitization processing via our internal Laboratory Information System. After personal privacy information of the patients was deleted, these data were utilized for subsequent statistical analysis.

### Sample collection and preparation

To investigate the diagnostic effectiveness of IgLCs in patients suspected of CHD, a total of 486 samples were collected. At the same time, we also collected other clinical information of these patients from the case system, including basic information such as age, sex, and other test data. All participants were strictly instructed to fast for 12 h and avoid strenuous activities prior to blood sampling to ensure that the levels of the collected blood components would be stable and could be accurately measured.

After collection, the blood samples were preprocessed within 2 h to ensure they would be fresh and suitable for further analysis. During processing, the samples were centrifuged at a constant temperature of 4 °C and a speed of 3000 rpm for 10 min, allowing adequate separation of the blood components. The rigorously processed blood samples were then submitted for TLC and FLC detection within 2 h after preprocessing.

### TLC and FLC detection and calculation of the CHI

TLCs and FLCs were detected and their concentrations measured on a Siemens BN II Automated Protein Analyzer (Siemens Healthcare Diagnostics Ltd., Erlangen, Germany). In accordance with the manufacturer’s instructions, the instrument was calibrated with quality control samples prior to testing the study samples. The plasma samples were tested for measuring the concentrations TLC κ, TLC λ, FLC κ, and FLC λ using the reagents provided by the manufacturer, and the data were recorded for further analysis. The ratios TLC κ/TLC λ and FLC κ/FLC λ are automatically generated by the BN II Automated Protein Analyzer. Drawing upon the biological observation that the ratio of κ-type to λ-type free light chains (FLC κ/FLC λ) remains relatively stable in the peripheral blood of healthy individuals, we devised and calculated the CHI. This indicator is defined as the ratio of the proportion of κ-type free light chains to total κ-type light chains (FLC κ/TLC κ) divided by the proportion of λ-type free light chains to total λ-type light chains (FLC λ/TLC λ), expressed as (FLC κ/TLC κ) / (FLC λ/TLC λ). This novel approach seeks to quantify the relative variations in these proportions, with the goal of uncovering potential associations between these changes and coronary health status.

### Establishment and selection of machine learning models

We have fully leveraged the powerful capabilities of the DxAI platform (https://www.xsmartanalysis.com) to complete all analytical work related to ML models. A binary logistic regression model was constructed using all the collected clinical data to conduct univariate analysis. The relationship between each feature and CHD as well as the control group was observed. A p-value less than 0.05 was considered statistically significant, and these significant features were then utilized for subsequent machine learning model construction.

We employed a resampling-based 5-fold cross-validation training/validation framework to systematically evaluate the average AUC scores of various machine learning models across different data partitions, providing a comprehensive assessment of their performance. Specifically, we allocated 20% of the data as the validation set to assess model generalizability. We employed nine distinct machine learning algorithms to generate the classifier: XGBoost, logistic regression (LR), Light GBM, random forest (RF), adaptive boosting (AdaBoost), Gaussian naive Bayes (GNB), multilayer perceptron (MLP), Decision Tree (DT) classification, and k-nearest neighbors (KNN). The model performance was evaluated using Receiver Operating Characteristic (ROC) curves, and the Area Under the Curve (AUC) was calculated for each model. Furthermore, the DeLong test was performed to assess whether the differences between the performances of the different models were statistically significant (*p* < 0.05). The model that demonstrated the best performance in both the training set and the validation set was selected as the subsequent research object. After selecting the best-performing ML model, we utilized the ROC curves generated from 10-fold cross-validation to evaluate the model’s classification performance in both the training and validation sets. We also employed learning curves (LCs) to assess the model’s performance trends as the number of training samples increases. Furthermore, calibration curves (CCs) were used to evaluate the consistency between the model’s predicted probabilities and the actual sample probabilities. Lastly, decision curve analysis (DCA) was applied to quantify the clinical benefit of the model. We did not perform an in-depth tuning of the XGBoost model. This was primarily due to our aim of maintaining the simplicity of the model and mitigating the risk of overfitting that could arise from excessive tuning. Furthermore, given the limitations of time and computational resources, we chose to proceed with the XGBoost model using its default parameters for subsequent analysis.

### Data analysis

Baseline data analysis as well as the construction of binary logistic regression and machine learning models were conducted on the DxAI platform (https://www.xsmartanalysis.com). All data analyses were performed via R version 4.2.3 and Python version 3.11.4. For single-factor regression analysis, the statsmodels Python package version 0.11.1 was utilized. The classification models were implemented in Python for subsequent performance comparison: eXtreme Gradient Boosting (XGBoost) was implemented with xgboost 2.0.1, Light Gradient Boosting Machine (LightGBM) with lightgbm version 3.2.1, and the other methods with scikit-learn 1.1.3. Apart from the construction of predictive models, all other statistical analysis results were generated via the CNSknowall platform (https://cnsknowall.com), a comprehensive web service for biomedical data analysis and visualization.

### Statistics

The data are presented and the mean and standard deviation (SD) of each group. Comparisons of sex distributions between the control and CHD groups were conducted with the chi-square test, whereas the other indicators were compared with the t test or Mann‒Whitney U test. When comparing data among multiple groups, we first employed ANOVA; if it yielded significant differences, we then proceeded to use Fisher’s least significant difference (LSD) method for a more detailed, pairwise comparison. A *p* value < 0.05was considered to indicate statistical significance.

## Results

### Subsection describing the patient recruitment

**Fig. 1:**
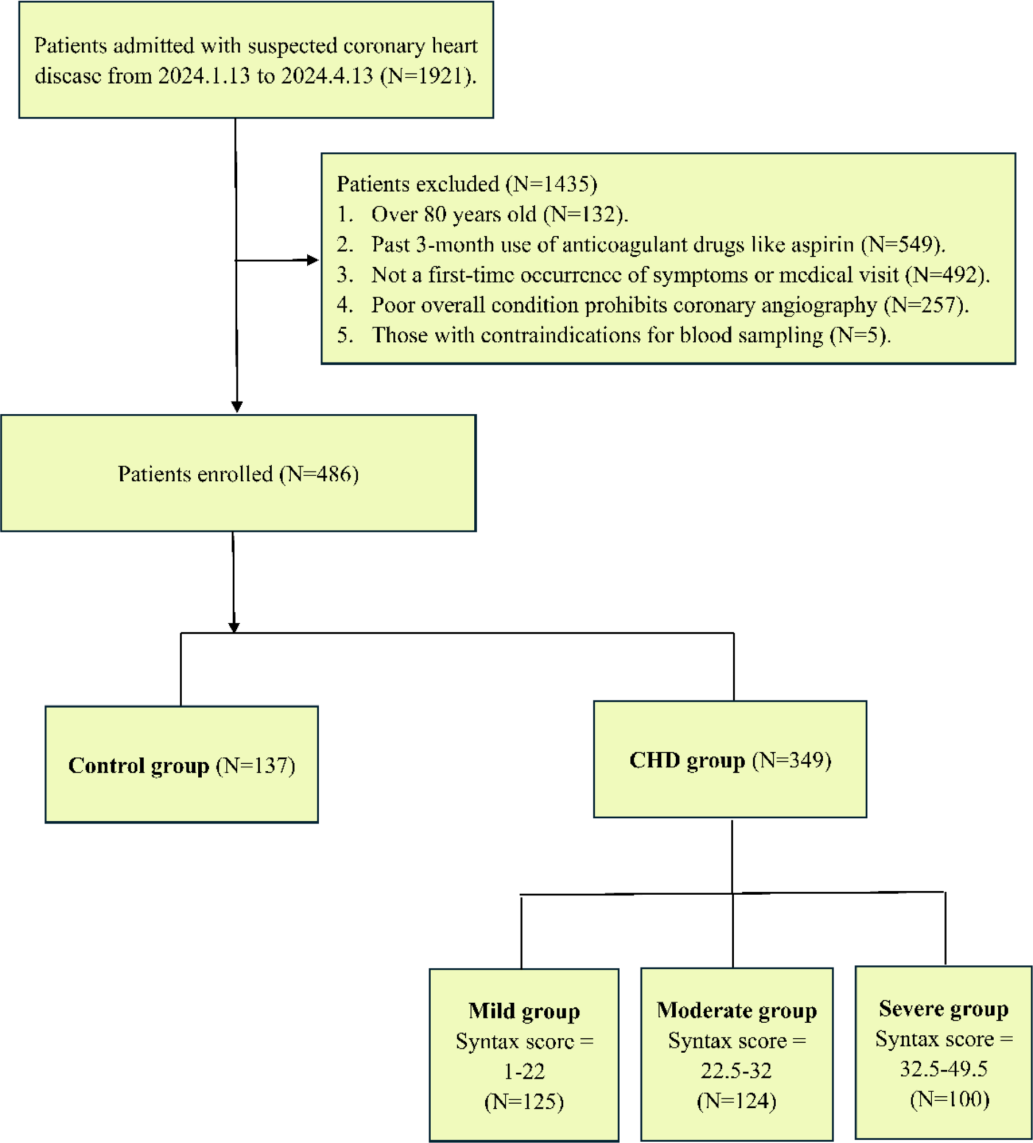
Details of the sample inclusion criteria and grouping procedure. After initial screening, the 486 included samples were divided into control and CHD groups. The CHD group was classified into mild, moderate, and severe lesion groups via the SYNTAX scoring system

Initially, 1921 samples and patient data were collected. After rigorous screening, 1435 were excluded, leaving 486 final cases. Exclusion reasons included age > 80 (*N* = 132), recent anticoagulant use (*N* = 549), ineligibility for angiography, and other factors (*N* = 5). Based on angiography, the 486 cases were divided into Control (*N* = 137) and CHD (*N* = 349) groups. Based on coronary angiography and SYNTAX scores, the CHD group was divided into mild (*N* = 125, SYNTAX 1–22), moderate (*N* = 124, SYNTAX 22.5–32), and severe (*N* = 100, SYNTAX 32.5–49.5) lesions for modeling to assess CHI’s distinction among CHD severities (Fig. 1). Descriptive stats were computed (Table 1), with BMI having the highest missing rate (23.66%), but most below 16%. No imputation was done due to low missing rates’ minimal impact on analysis.

**Table 1:** Descriptive statistics of the characteristics of the sample

Var	Mean	Med	Q3	SD	Min	Max	MissR%
CHI	0.51	0.52	0.58	0.11	0.11	0.81	0.00
SYNTAX	17.71	21.00	30.50	14.84	0.00	49.50	0.00
Age	60.50	61.00	68.00	10.85	1.00	89.00	0.00
BMI	25.06	23.33	26.67	17.72	14.17	259.33	23.66
SBP	136.75	134.00	147.50	49.28	0.01	1090.00	5.56
DBP	81.18	80.00	90.00	12.40	47.00	122.00	5.56
Tro	3.54	0.01	0.58	14.07	0.01	206.00	9.465
BNP	956.03	90.49	473.28	3456.57	3.42	39600.00	10.29
EF	0.58	0.59	0.63	0.13	0.18	1.18	9.67
EDV	117.32	108.50	136.75	43.957	25.00	368.00	9.88
ESV	45.74	41.00	54.00	38.50	2.11	433.00	10.08
SV	65.13	66.00	83.00	46.13	0.01	779.00	12.35
CO	25.97	5.71	7.5100	88.73	0.82	500.00	15.43
D-D	443.61	267.77	425.38	860.30	0.00	13649.18	12.55
HDL	1.09	1.03	1.24	0.41	0.36	5.14	8.03
LDL	2.90	2.92	3.41	0.90	0.67	6.59	8.03
TC	4.56	4.55	5.25	1.48	0.42	14.48	8.03
TG	1.98	1.43	2.23	2.24	0.17	23.91	8.03
LYM#	1.85	1.78	2.31	0.83	0.16	5.86	10.50
NEU#	5.57	4.63	6.91	3.12	0.17	24.97	10.50
NLR	4.18	2.59	4.13	5.78	0.10	89.13	10.50
LYM%	0.25	0.25	0.33	0.10	0.03	0.73	11.11
Myo	91.19	26.68	57.52	149.21	5.00	500.00	10.70
CKMB	12.70	3.00	4.27	24.55	0.22	100.00	10.50

BMI: Body mass index. SBP: Systolic blood pressure. DBP: Diastolic blood pressure. Tro: Troponin. BNP: Brain natriuretic peptide. EF: Ejection fraction. EDV: End-diastolic volume. ESV: End-systolic volume. SV: Stroke volume. CO: Cardiac output. D-D: D-Dimer. HDL: High-density lipoprotein. LDL: Low-density lipoprotein. TC: Total cholesterol. TG: Triglyceride. LYM#: Absolute lymphocyte count. NEU#: Absolute neutrophil count. NLR: Neutrophil-to-lymphocyte ratio. LYM%: Lymphocyte percentage. Myo: Myoglobin. CK-MB: Creatine kinase-MB isoenzyme. Q3: Third quartile

### CHD patients present with unique baseline characteristics and a lower CHI than controls

**Fig. 2:**
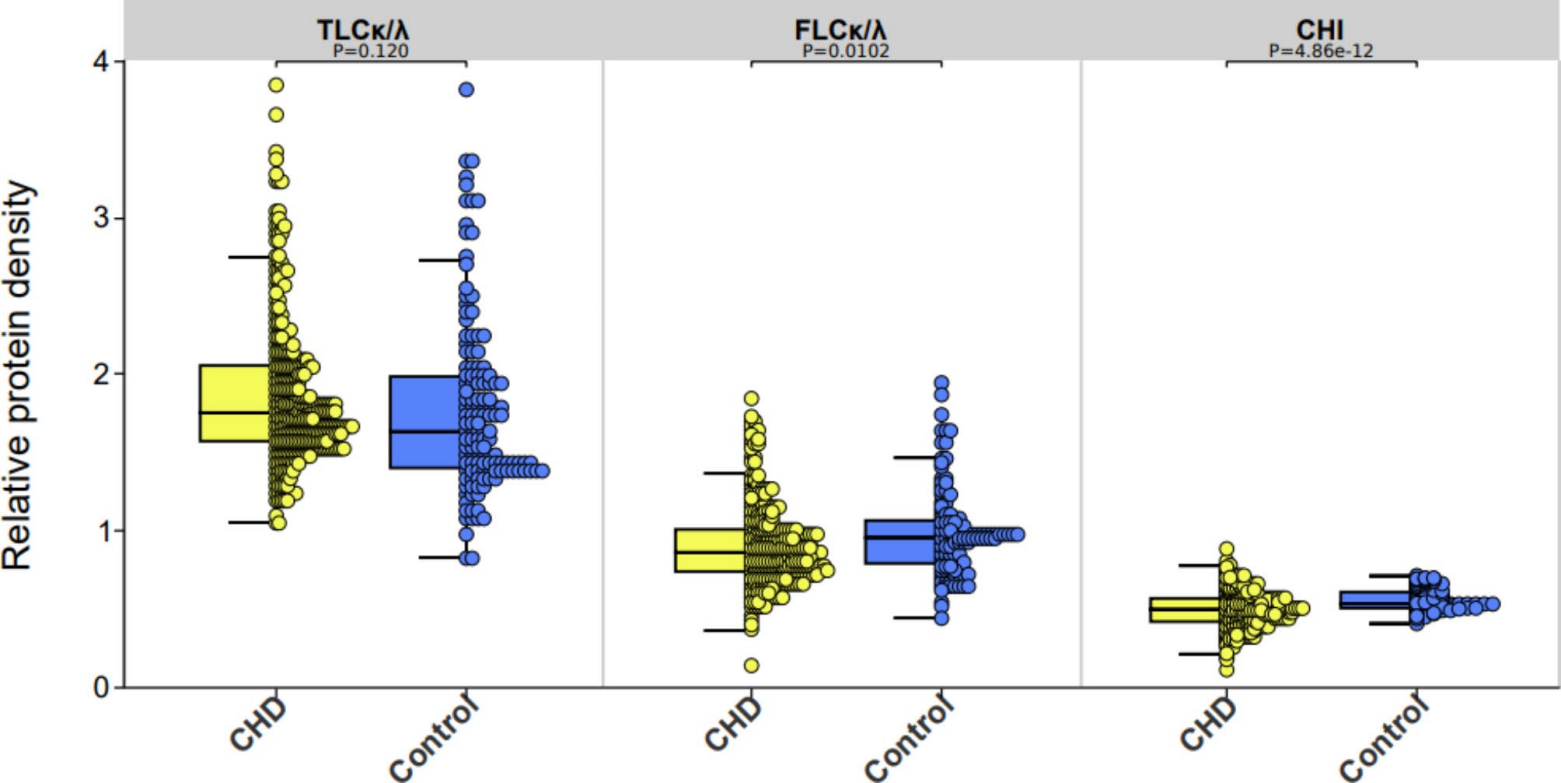
CHD patients show distinct features and lower CHI than controls. Comparison of the classical diagnostic indicators as well as the novel CHI between the control group and the CHD group

To verify the overall disparities between volunteers in the two groups categorized based on coronary angiography data, we thoroughly compared the baseline characteristics of the CHD group with those of the control group. The outcomes revealed differences (*p*<0.05) in multiple crucial physiological and biochemical parameters between the two groups. Notably, CHD patients presented with higher levels of various biomarkers, such as age, troponin, BNP, D-dimer level (DD), neutrophil count (NEU#), neutrophil-to-lymphocyte ratio (NLR), myoglobin, and creatinine kinase-MB (CK-MB), and lower diastolic blood pressure, lymphocyte count (LYM#), and lymphocyte percentage (LYM%) (Table 2). After comparing the control group with the CHD group, we found that the ratios of TLCκ/TLCλ (*p* = 0.120) was not significantly different, whereas the FLCκ/FLCλ ratio (*p* = 0.0102) demonstrated a statistically significant difference (Fig. 2). However, when we convert the differences in IgLCs into CHI, significant statistical differences are shown between the CHD group and the Control group (*p*<0.01).

**Table 2:** Baseline characteristics of the control and CHD groups

Characteristics	Control group (Median [IQR])	CHD group (Median [IQR])	*p* value
**Sex**,** N (%)**	**Female 56 (40.876)**	**106 (30.372)**	**0.027**
	**Male 81 (59.124)**	**243 (69.628)**	
**CHI**	**0.532 [0.505**,** 0.607]**	**0.497 [0.418**,** 0.567]**	**0.001**
**SYNTAX Score**	**0.0 [0.0**,** 0.500]**	**25.0 [17.0**,** 33.500]**	**0.001**
**Age**	**57.0 [51.0**,** 63.0]**	**62.0 [55.0**,** 68.0]**	**0.001**
BMI	22.333 [20.0, 26.0]	23.333 [20.0, 26.667]	0.426
SBP	135.0 [120.0, 150.0]	132.0 [122.0, 147.0]	0.826
DBP	85.0 [74.0, 91.0]	80.0 [72.0, 90.0]	0.09
**Tro**	**0.010 [0.010**,** 0.010]**	**0.059 [0.010**,** 1.830]**	**0.001**
**BNP**	**34.680 [10.0**,** 130.980]**	**153.010 [32.790**,** 693.180]**	**0.001**
EF	0.590 [0.470, 0.630]	0.590 [0.520, 0.630]	0.374
**EDV**	**103.0 [79.0**,** 131.0]**	**115.0 [88.0**,** 139.0]**	**0.024**
**ESV**	**35.0 [26.0**,** 50.0]**	**44.0 [29.0**,** 58.0]**	**0.017**
SV	65.0 [58.0, 85.0]	67.0 [48.0, 83.0]	0.119
CO	5.410 [4.710, 7.210]	6.010 [4.710, 7.810]	0.08
**DD**	**238.750 [178.760**,** 363.710]**	**279.900 [185.410**,** 469.310]**	**0.025**
**HDL**	**1.080 [0.920**,** 1.310]**	**1.0 [0.880**,** 1.190]**	**0.005**
LDL	3.0 [2.260, 3.350]	2.920 [2.320, 3.520]	0.241
TC	4.550 [3.690, 4.850]	4.550 [3.740, 5.400]	0.056
**TG**	**1.280 [0.930**,** 1.980]**	**1.500 [1.050**,** 2.310]**	**0.014**
**LYM #**	**1.960 [1.440**,** 2.470]**	**1.670 [1.210**,** 2.150]**	**0.001**
**NEU#**	**3.940 [3.330**,** 6.260]**	**4.890 [3.820**,** 7.300]**	**0.001**
**NLR**	**2.075 [1.484**,** 2.849]**	**2.860 [1.990**,** 5.180]**	**0.001**
**LYM %**	**0.300 [0.240**,** 0.360]**	**0.240 [0.160**,** 0.300]**	**0.001**
**Myo**	**21.050 [13.700**,** 36.190]**	**28.770 [19.590**,** 101.0]**	**0.001**
**CKMB**	**3.0 [3.0**,** 3.0]**	**3.0 [3.0**,** 7.550]**	**0.001**

IQR: Interquartile range. BMI: Body mass index. SBP: Systolic blood pressure. DBP: Diastolic blood pressure. Tro: Troponin. BNP: Brain natriuretic peptide. EF: Ejection fraction. EDV: End-diastolic volume. ESV: End-systolic volume. SV: Stroke volume. CO: Cardiac output. D-D: D-Dimer. HDL: High-density lipoprotein. LDL: Low-density lipoprotein. TC: Total cholesterol. TG: Triglyceride. LYM#: Absolute lymphocyte count. NEU#: Absolute neutrophil count. NLR: Neutrophil-to-lymphocyte ratio. LYM%: Lymphocyte percentage. Myo: Myoglobin. CK-MB: Creatine kinase-MB isoenzyme. The DxAI platform performs a comprehensive intelligent baseline analysis that intelligently selects the appropriate analytical method based on the distribution of the samples, homogeneity of variance, and sample size. The chi-square test was used for sex, the t test for DBP, and the Mann‒Whitney U test for other features

### Feature selection for machine learning models

**Fig. 3:**
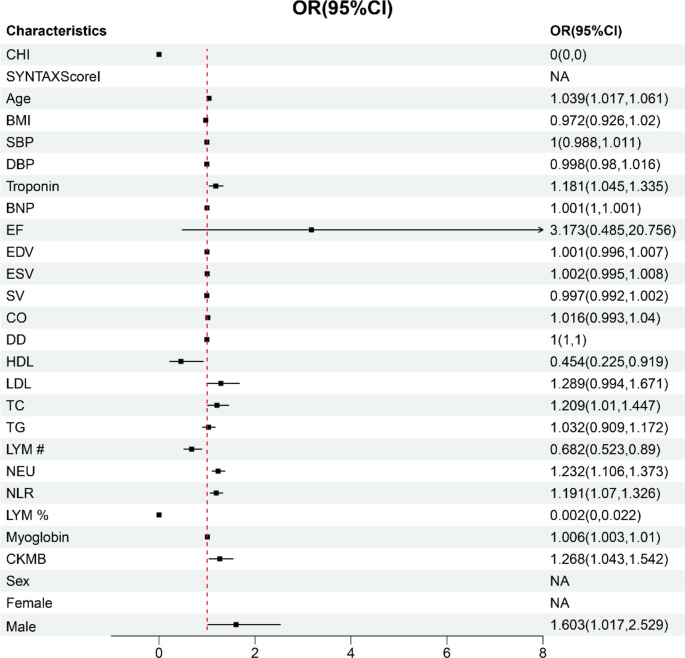
Logic regression is used for feature selection. Results of the univariate regression analysis used to screen for baseline characteristics significantly associated with CHD

We plan to explore the diagnostic value of CHI for CHD through the construction of ML models. In the initial stage, we have already conducted a univariate analysis using a binary logistic regression model for all baseline characteristics. Based on the significance level of *p* < 0.05 and the predictive strength suggested by odds ratios (ORs), we have identified a set of key indicators that will serve as the foundation for the subsequent construction of ML models. The results revealed that CHI, age, troponin, BNP, HDL, TC, LYM#, NEU#, NLR, LYM%, and myoglobin were significantly different between the groups (Fig. 3; Table 3). These indicators were then used to establish the diagnostic models.

**Table 3:** Univariate logistic regression analyses for selecting variables for model development

Characteristics	OR	95% CI	*p* value
Sex			
Female			
Male	1.603	[1.017, 2.529]	0.051
**CHI**	**0.0**	**[0.0**,** 0.0]**	**0.0**
**Age**	**1.039**	**[1.017**,** 1.061]**	**0.0**
BMI	0.972	[0.926, 1.02]	0.251
SBP	1.0	[0.988, 1.011]	0.935
DBP	0.998	[0.98, 1.016]	0.804
**Tro**	**1.181**	**[1.045**,** 1.335]**	**0.008**
**BNP**	**1.001**	**[1.0**,** 1.001]**	**0.003**
EF	3.173	[0.485, 20.756]	0.228
EDV	1.001	[0.996, 1.007]	0.651
ESV	1.002	[0.995, 1.008]	0.572
SV	0.997	[0.992, 1.002]	0.184
CO	1.016	[0.993, 1.04]	0.162
DD	1.0	[1.0, 1.0]	0.796
**HDL**	**0.454**	**[0.225**,** 0.919]**	**0.028**
LDL	1.289	[0.994, 1.671]	0.055
**TC**	**1.209**	**[1.01**,** 1.447]**	**0.038**
TG	1.032	[0.909, 1.172]	0.627
**LYM#**	**0.682**	**[0.523**,** 0.89]**	**0.005**
**NEU#**	**1.232**	**[1.106**,** 1.373]**	**0.0**
**NLR**	**1.191**	**[1.07**,** 1.326]**	**0.001**
**LYM%**	**0.002**	**[0.0**,** 0.022]**	**0.0**
**Myo**	**1.006**	**[1.003**,** 1.01]**	**0.001**
**CKMB**	**1.268**	**[1.043**,** 1.542]**	**0.017**

BMI: Body mass index. SBP: Systolic blood pressure. DBP: Diastolic blood pressure. Tro: Troponin. BNP: Brain natriuretic peptide. EF: Ejection fraction. EDV: End-diastolic volume. ESV: End-systolic volume. SV: Stroke volume. CO: Cardiac output. D-D: D-Dimer. HDL: High-density lipoprotein. LDL: Low-density lipoprotein. TC: Total cholesterol. TG: Triglyceride. LYM#: Absolute lymphocyte count. NEU#: Absolute neutrophil count. NLR: Neutrophil-to-lymphocyte ratio. LYM%: Lymphocyte percentage. Myo: Myoglobin. CK-MB: Creatine kinase-MB isoenzyme. OR: odds ratio. 95% CI: 95% confidence interval

### XGBoost classification model exhibits optimal performance

**Fig. 4:**
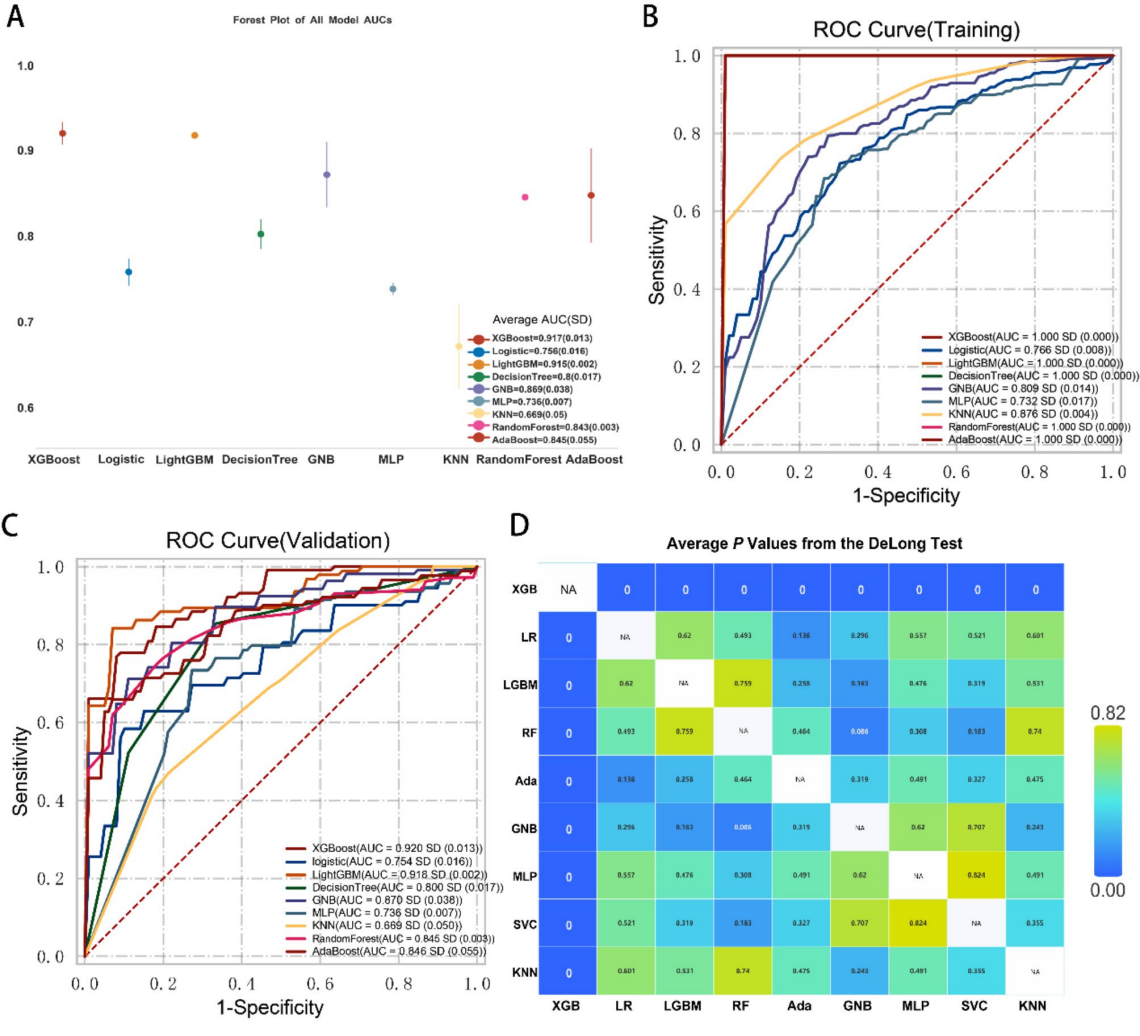
Screening of machine learning classification models. (**A**) The forest plot displays the results of ROC curve analysis for the CHD prediction models, with the data indicating the means (dots) and SDs (error bars) of the model AUCs. Through 5-fold cross-validation, XGBoost emerged as the top performer in the training set in terms of AUC. (**B**) Plots of the model ROC curves in the training set. (**C**) Plots of the model ROC curves in the validation set. The AUC value of the XGBoost classification model was the highest. (**D**) The DeLong test results indicate that the area under the ROC curve of the XGBoost model is significantly different from the AUCs of the other models

In our preliminary screening to identify the optimal machine learning model for classifying samples as CHD or control patients, we compared the performance of various models by calculating the average AUC of their ROC curves across both the training and validation sets. The model selection process was conducted on the DxAI platform, employing a five-fold cross-validation method with oversampling as the validation approach. The validation set comprised 20% of the total data, as mentioned in our methodology section, where we comprehensively evaluated nine machine learning models. Among these models, the XGBoost classifier model stood out, achieving an exceptional average AUC of 0.917 ± 0.023 (Fig. 4A). Individual AUC values for the training and validation sets are also provided to offer a more comprehensive view of the model’s performance. Using 5-fold cross-validation and variables such as CHI, Age, Troponin, BNP, HDL, TC, CKMB, Myoglobin, LYM#, NLR#, NEU, and LYM% as inputs, the XGBoost classifier model achieved nearly perfect performance in the training set, exhibiting an AUC of 1.0 ± 0.000, while metrics such as accuracy (0.996), sensitivity (0.995), and specificity (1.0) were all close to or reached 1.0 (Fig. 4B, Supplementary Table 1). In the validation set, the XGBoost classifier model maintained this good performance, with an AUC value of 0.920 ± 0.019; its accuracy (0.852), sensitivity (0.834), and specificity (0.940) in the validation set also demonstrated the good generalization ability of the XGBoost classifier model (Fig. 4C, Supplementary Table 2). The results of the DeLong test statistically validated the superiority of the XGBoost model over the other models in terms of AUC (*p* < 0.001) (Fig. 4D). After a comprehensive comparison of various metrics, including AUC, accuracy, specificity, and sensitivity, across both the training and validation sets for each model, the XGBoost model exhibited superior performance in both, with consistent results. Based on its performance in both sets, we can confidently conclude that the XGBoost classifier model is the optimal choice.

### The XGBoost model excels in identifying coronary heart disease patients

**Fig. 5:**
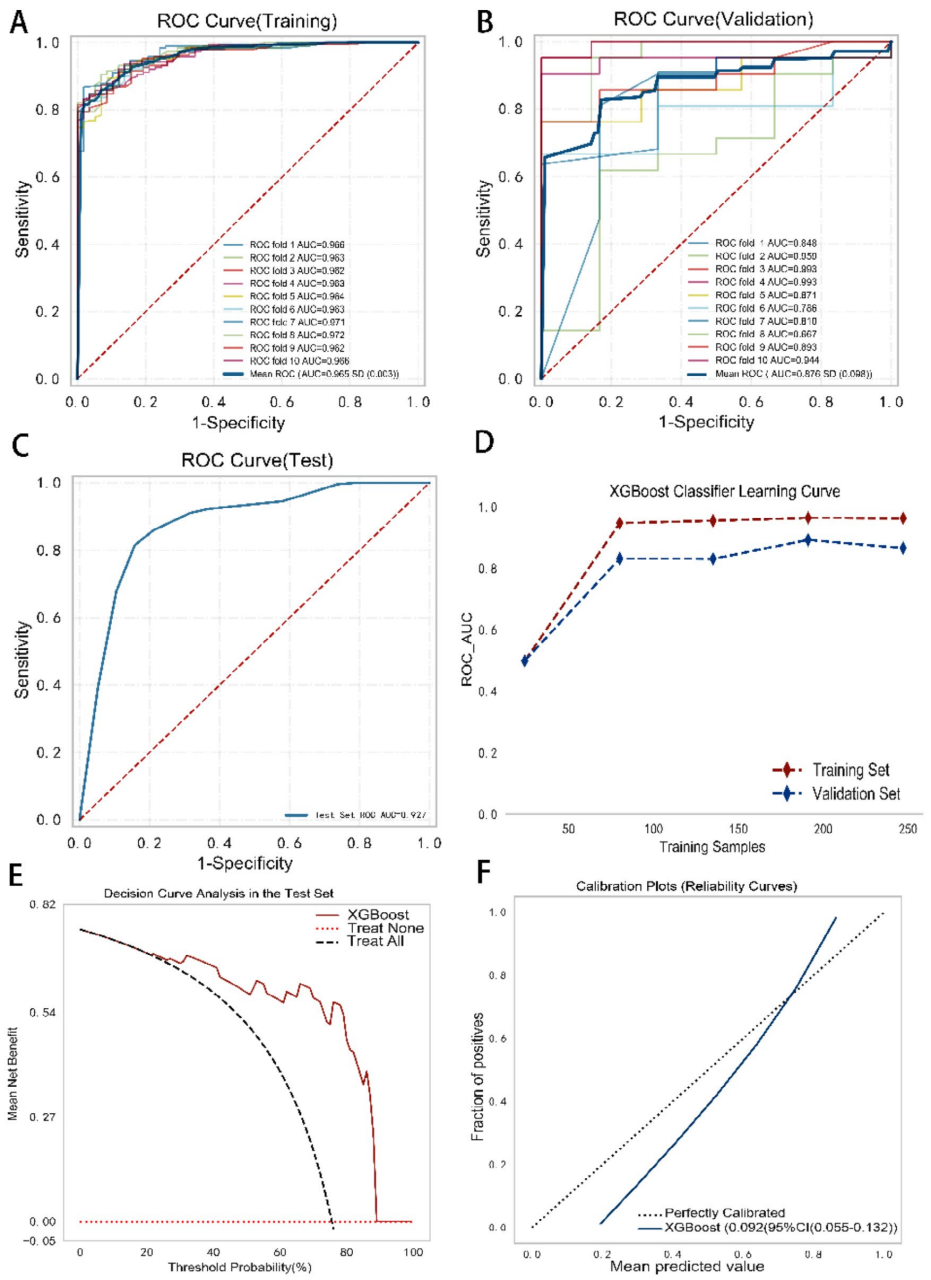
Performance evaluation of the XGBoost classification model. (**A**) 10-fold cross-validation ROC curves in the training set. (**B**) 10-fold cross-validation ROC curves in the validation set. (**C**) ROC curve in the test set. (**D**) The learning curve shows that model performance in both the training set and the validation set improves steadily and that the difference between them remains stable, showing that the model neither overfits nor underfits the data and is able to effectively learn from the training data and generalize to the validation data. (**E**) The decision curve highlights the model’s excellent performance across multiple thresholds, indicating that it could help guide clinical decision-making. (F) The calibration curve shows close alignment between the predicted and actual probabilities, demonstrating the accuracy of the model’s predictive performance

After initially selecting the optimal model, we conducted a more thorough evaluation of the XGBoost classification model’s efficacy in diagnosing CHD patients (with respect to the actual patient outcomes through calibration curve analysis) and its potential benefits for clinical practice (through decision curve analysis). Specifically, the training, validation, and tests comprised 65%, 20%, and 15% of the original data; we trained XGBoost model with the training set through 10-fold cross-validation, then assessed model performance in the test set. Subsequently, in the training set, validation set, and test set, the XGBoost model achieved AUCs of 0.949 ± 0.003, 0.882 ± 0.088, and 0.925, respectively, while the accuracies were 0.858, 0.803, and 0.878, respectively (Fig. 5A, B and C, Supplementary Table 3). Although the AUC in the validation set did not surpass the training set AUC, the XGBoost model clearly achieved a successful fit, thereby validating its suitability for classification in this specific dataset (Fig. 5D).

The results of decision curve (Fig. 5E) and calibration curve (Fig. 5F) analyses also indicated that the XGBoost model had good clinical utility and calibration, respectively, in identifying patients with CHD. Decision curve analysis demonstrated that the model performed exceptionally well across multiple intervention thresholds, offering net benefits over “treat all” and “treat none” approaches and providing effective guidance for clinical decision-making. Calibration curve analysis indicated that the model’s predicted probabilities align well with the actual observed outcomes, with the red (fitted) line closely resembling the reference (diagonal) line, without significant overestimation or underestimation of risks. The XGBoost model exhibits not only exceptional effectiveness but also substantial clinical practicability and reliability when it comes to diagnosing CHD patients.

### CHI decreases with severity of CHD patients

**Fig. 6:**
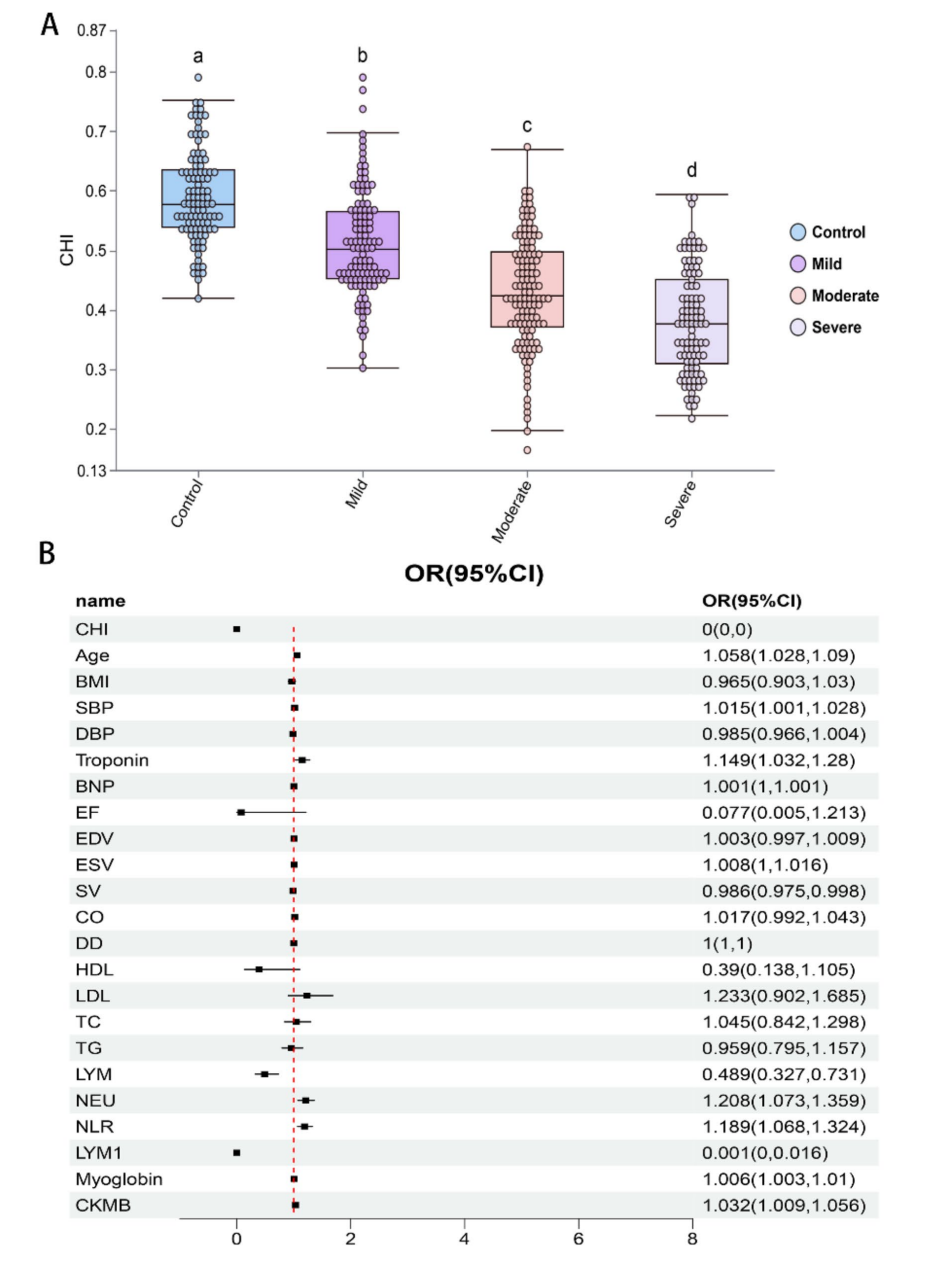
CHI decreases with severity of CHD patients. (**A**) As the severity of CHD increases, the CHI decreases, as depicted in a set of box plots. (**B**) The forest plot displays the ROC curve results for the CHD prediction models, with dots and error bars indicating the means and SDs of the AUC. Through 2-fold cross-validation, XGBoost emerged as the top performer in the training set according to the AUC

Beyond merely rapidly identifying CHD patients within high-risk populations, a precise assessment of the severity of an individual patient’s disease is paramount for crafting personalized treatment strategies and enhancing patient outcomes. Leveraging the SYNTAX scoring system, we have systematically divided the CHD group samples into mild, moderate, and severe categories. Our preliminary analysis unveiled striking disparities in the Coronary Heart Index (CHI) across these four groups, notably observing a decline in CHI as the SYNTAX score escalated, with the control group exhibiting the highest CHI values (Fig. 6A).

Subsequently, we embarked on developing a methodology to distinguish severe CHD patients from healthy controls. Our baseline data analysis disclosed significant variations in a multitude of indicators between these two groups, as detailed in Table 4. Employing univariate regression analysis, we pinpointed a total of 12 salient features for subsequent model selection and construction (Fig. 6B; Table 5). Following examination, we harnessed these screened variables to formulate classification models utilizing nine distinct algorithms tailored for the CHD versus control classification task.

**Table 4:** Comparison of baseline characteristics between the control and severe CHD groups

Characteristics	Control group (Median [IQR])	Severe CHD group (Median [IQR])	*p* value
**Sex**,** N (%)**			
**Female**	**56 (40.876)**	**27 (27.0)**	
**Male**	**81 (59.124)**	**73 (73.0)**	**0.027**
**CHI**	**0.545 [0.507**,** 0.607]**	**0.390 [0.344**,** 0.468]**	**< 0.001**
**Age**	**58.0 [52.0**,** 69.0]**	**65.0 [59.0**,** 70.0]**	**< 0.001**
BMI	22.333 [20.0, 26.0]	23.333 [20.0, 26.667]	0.765
SBP	136.0 [120.0, 150.0]	140.0 [124.0, 154.0]	0.142
DBP	80.0 [70.0, 90.0]	80.0 [75.0, 90.0]	0.777
**Troponin**	**0.010 [0.010**,** 0.010]**	**0.065 [0.010**,** 1.540]**	**< 0.001**
**BNP**	**10.990 [10.0**,** 57.630]**	**182.920 [49.840**,** 1095.460]**	**< 0.001**
**EF**	**0.590 [0.470**,** 0.630]**	**0.550 [0.450**,** 0.620]**	**0.038**
**EDV**	**103.0 [79.0**,** 131.0]**	**119.0 [87.0**,** 148.0]**	**0.040**
**ESV**	**35.0 [26.0**,** 50.0]**	**49.0 [34.0**,** 74.0]**	**0.001**
SV	65.0 [58.0, 85.0]	68.0 [48.0, 83.0]	0.210
CO	5.410 [4.710, 7.210]	5.610 [4.610, 7.010]	0.809
**DD**	**238.750 [178.760**,** 363.710]**	**366.0 [249.980**,** 521.690]**	**< 0.001**
**HDL**	**1.080 [0.920**,** 1.310]**	**1.0 [0.870**,** 1.140]**	**0.004**
LDL	3.0 [2.260, 3.350]	2.870 [2.240, 3.680]	0.497
TC	4.550 [3.690, 4.850]	4.400 [3.490, 5.290]	0.875
TG	1.280 [0.930, 1.980]	1.390 [1.060, 2.270]	0.168
**LYM#**	**1.960 [1.440**,** 2.470]**	**1.520 [1.110**,** 2.030]**	**< 0.001**
**NEU#**	**3.940 [3.330**,** 6.260]**	**4.810 [3.670**,** 7.480]**	**0.003**
**NLR**	**2.075 [1.484**,** 2.849]**	**3.0 [2.040**,** 5.343]**	**< 0.001**
**LYM%**	**0.300 [0.240**,** 0.360]**	**0.220 [0.150**,** 0.290]**	**< 0.001**
**Myoglobin**	**21.050 [13.700**,** 36.190]**	**31.290 [20.570**,** 131.940]**	**< 0.001**
**CKMB**	**3.0 [3.0**,** 3.0]**	**3.0 [3.0**,** 8.400]**	**< 0.001**

IQR: Interquartile range. BMI: Body mass index. SBP: Systolic blood pressure. DBP: Diastolic blood pressure. BNP: Brain natriuretic peptide. EF: Ejection fraction. EDV: End-diastolic volume. ESV: End-systolic volume. SV: Stroke volume. CO: Cardiac output. D-D: D-Dimer. HDL: High-density lipoprotein. LDL: Low-density lipoprotein. TC: Total cholesterol. TG: Triglyceride. LYM#: Absolute lymphocyte count. NEU#: Absolute neutrophil count. NLR: Neutrophil-to-lymphocyte ratio. LYM%: Lymphocyte percentage. CK-MB: Creatine kinase-MB isoenzyme. Comprehensive intelligent baseline analysis intelligently selects the most suitable analytical method based on the distribution of samples, homogeneity of variance, and sample size. For this dataset, all indicators were analyzed with the Mann‒Whitney U test

**Table 5:** Univariate logistic regression selects clinical data for CHD severity classification model development

Characteristics	OR	95% CI	*p* value
**CHI**	**0.0**	**[0.0**,** 0.0]**	**< 0.001**
**Age**	**1.058**	**[1.028**,** 1.09]**	**< 0.001**
BMI	0.965	[0.903, 1.03]	0.284
**SBP**	**1.015**	**[1.001**,** 1.028]**	**0.031**
DBP	0.985	[0.966, 1.004]	0.130
**Troponin**	**1.149**	**[1.032**,** 1.28]**	**0.011**
**BNP**	**1.001**	**[1.0**,** 1.001]**	**0.002**
EF	0.077	[0.005, 1.213]	0.068
EDV	1.003	[0.997, 1.009]	0.331
ESV	1.008	[1.0, 1.016]	0.056
**SV**	**0.986**	**[0.975**,** 0.998]**	**0.017**
CO	1.017	[0.992, 1.043]	0.194
DD	1.0	[1.0, 1.0]	0.691
HDL	0.39	[0.138, 1.105]	0.076
LDL	1.233	[0.902, 1.685]	0.190
TC	1.045	[0.842, 1.298]	0.689
TG	0.959	[0.795, 1.157]	0.661
**LYM#**	**0.489**	**[0.327**,** 0.731]**	**< 0.001**
**NEU**	**1.208**	**[1.073**,** 1.359]**	**0.002**
**NLR**	**1.189**	**[1.068**,** 1.324]**	**0.002**
**LYM%**	**0.001**	**[0.0**,** 0.016]**	**< 0.001**
**Myoglobin**	**1.006**	**[1.003**,** 1.01]**	**< 0.001**
**CKMB**	**1.032**	**[1.009**,** 1.056]**	**0.007**

OR: Odds ratio. 95% CI: 95% confidence interval

### XGBoost also accurately identifies severe coronary heart disease patients

**Fig. 7:**
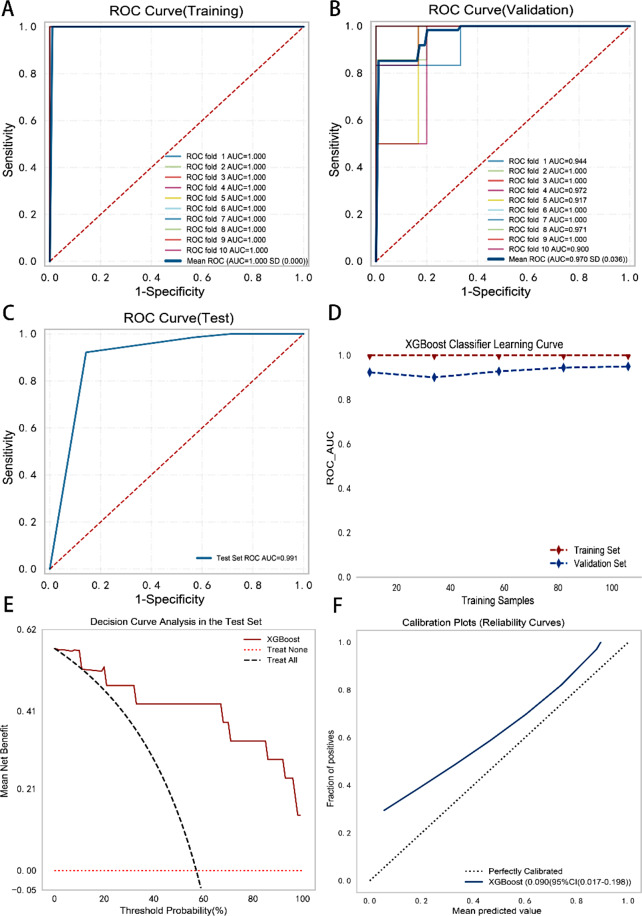
XGBoost model identifies patients with severe CHD. (**A**) 10-fold cross-validation ROC curves in the training set. (**B**) 10-fold cross-validation ROC curves in the validation set. (**C**) ROC curve in the test set. (**D**) The learning curves exhibit similar trends between the training and validation sets with small differences, indicating strong generalization capabilities and near-optimal performance. (**E**) Decision curve analysis reveals that the model outperforms alternative treatment strategies and is clinically effective. (**F**) The calibration curve reveals the model’s overconfidence in predicting severe CHD

Through rigorous comparisons, XGBoost again emerged as the optimal model for distinguishing between the control and severe groups (Supplementary Fig. 1, Supplementary Table 4, Supplementary Table 5). We first employed 10-fold cross-validation and conducted ROC curve analysis in the training and validation sets, achieving average AUC values of 1.0 ± 0.000 and 0.97 ± 0.036, respectively, indicating that the XGBoost model demonstrated excellent performance in predicting severe CHD patients (Fig. 7A and B); this performance was validated in the test set, in which ROC curve analysis yielded an AUC of 0.991 (Fig. 7C).

The learning curves showed similar trends between the training and validation sets, with small differences between them, indicating strong generalizability. As the sample size increased, the curves remained stable (Fig. 7D). Decision curve analysis revealed that the model provided greater net clinical benefits than strategies assuming all or none of the patients had severe CHD, confirming its clinical utility (Fig. 7E). Calibration curve analysis showed that the model was overconfident in predicting severe CHD, with a Brier score of 0.09 (Fig. 7F); nevertheless, considering the F1 score (0.857), accuracy (Table 6), and other evaluation metrics such as the AUC, we concluded that the model’s predictions are relatively trustworthy.

**Table 6:** Comprehensive summary of the performance metrics of the model in the three sets

Set	AUC	Cutoff	Acc	Sen	Spec	PPV	NPV	F1
Training	1.000	0.770	0.991	0.982	1.000	1.000	0.981	0.991
Validation	0.970	0.770	0.907	0.869	0.943	0.951	0.878	0.905
Test	0.991	0.669	0.857	0.75	1.0	1.0	0.75	0.857

AUC: Area under the curve. Acc: Accuracy. Sen: Sensitivity. Spec: Specificity. PPV: Positive predictive value. NPV: Negative predictive value. The original data were divided into training, validation, and test sets at proportions of 65%, 20%, and 15%, respectively

### Development of online prediction tool for external validation

**Fig. 8:**
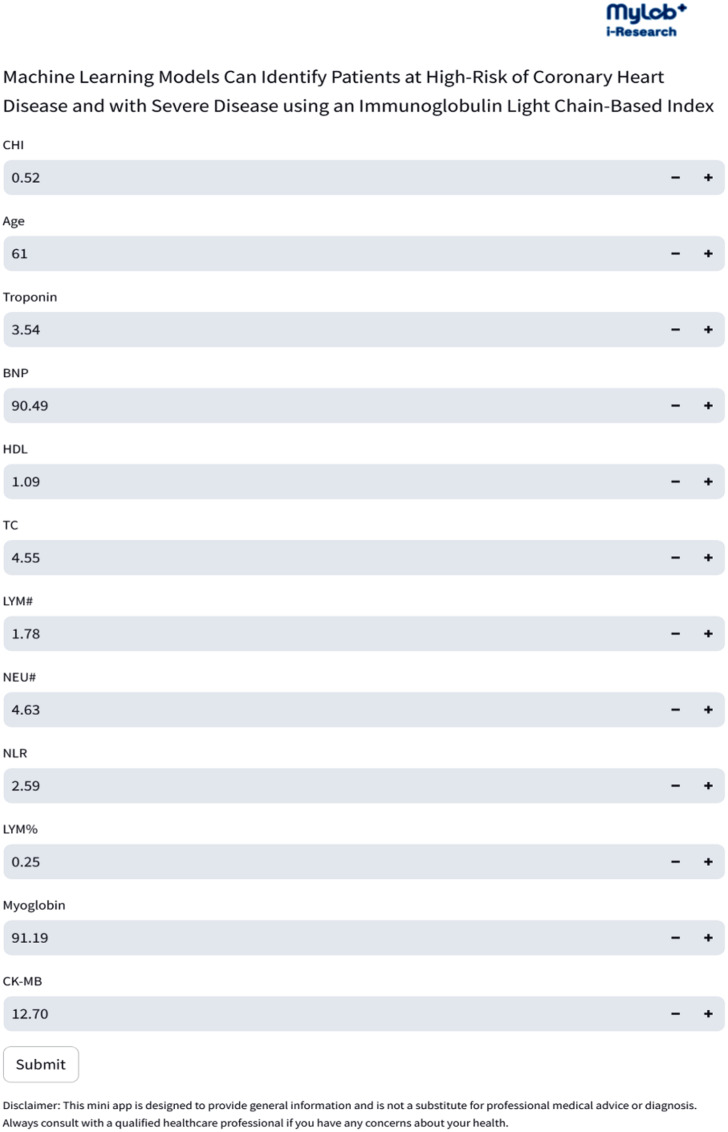
Development of online prediction tool. The visualization of the prediction model through Roche i-Research platform

To obtain a wider range of external validation data, we have developed and presented an online web tool through the Roche i-Research platform. This tool generates predictive models based on current algorithms and can predict the risk of a positive result by setting parameters (Fig. 8A). Upon inputting relevant parameters, the tool calculates the probability of a subject having CHD (https://jg4dbugczt6zmxqjt7asg4.streamlit.app/). Subsequently, we conducted a preliminary external validation of the tool to assess its practical application effects. Samples were collected according to a unified standard from the Clinical Laboratory of Lequn Branch, The First Hospital of Jilin University, totaling 59 blood samples from patients with CHD. According to the SYNTAX score, 12 of the cases belong to severely complex lesions. By comparing the prediction results with clinical diagnoses, we found that the accuracy of the tool in diagnosing coronary heart disease was 86.44%, and the accuracy for diagnosing patients with severe lesions reached 100%, demonstrating good clinical application value and accuracy (Supplementary Table 6).

## Discussion

Despite significant advancements in the diagnosis and treatment of CHD in recent decades, there are still notable deficiencies in routinely screening high-risk populations to accurately identify asymptomatic CHD patients. Moreover, in line with the growing demand for precision medicine, there remains a gap in utilizing blood samples to rapidly and conveniently differentiate CHD patients based on varying degrees of severity, thereby guiding clinicians to adopt appropriate treatment strategies. Recent studies have uncovered potential associations between IgLCs and CHD, yet conventional diagnostic markers such as the FLCκ/λ have proven ineffective for direct application in CHD screening and diagnosis, necessitating further exploration and optimization. In this study, we developed a novel index based on IgLCs called the CHI, which could not only aid in the early diagnosis of CHD but also precisely identified patients with severe CHD. By including it as an input to an XGBoost classification model, furthermore, the model demonstrates exceptional accuracy and sensitivity, ensuring its reliability in clinical applications.

Owing to the long-term progression of coronary artery atherosclerosis, CHD often develops subtly, with patients potentially remaining asymptomatic for extended periods. Indeed, the proportion of asymptomatic CHD patients even exceeds that of symptomatic patients [22]. Current diagnostic gold standards, which often involve invasive procedures and potential radiation risks, limit their applicability in screening a broader population of asymptomatic individuals with potential CHD. Prior studies have attempted to explore the potential correlation between the presence of FLCs in the blood and the development of CHD. One study revealed that there was no significant difference in the levels of FLCs between patients with ST-elevation myocardial infarction (STEMI) and healthy controls [19]. However, unlike in that study, the indicator we used in our research was the FLC κ/λ ratio, which is widely adopted in the diagnosis of multiple myeloma [23]. Our research showed no significant difference in TLCκ/TLCλ ratio between control and CHD groups, but a statistically significant difference was found in FLCκ/FLCλ ratio. This suggests that, while TLCκ/TLCλ changes may not be substantial in CHD, FLCκ/FLCλ alterations may indicate specific pathological processes. However, a previous study, using a different population or methodology, found no significant difference in the FLCκ/FLCλ between healthy controls and patients with type 2 diabetes, stable angina, non-STEMI, or STEMI. Interestingly, that study classified participants based on a median FLC κ/λ ratio of 0.63, and those with a κ/λ ratio above 0.63 were more likely to exhibit improved cardiac function over time [20]. This suggests that, although the FLCκ/FLCλ ratio may not differentiate between healthy and diseased states in all studies, it may still hold prognostic value in certain subpopulations. Therefore, we should concentrate on developing an assessment system for IgLCs that is grounded in FLCs and offers greater universality and precision.

Based on the information we have gathered, the ratio of kappa and lambda free light chains (FLC κ/FLC λ) in peripheral blood remains relatively stable under healthy conditions [14]. However, in disease states, the balance between the production and clearance of light chains is disrupted, leading to excessive accumulation of a specific light chain type in the blood [11]. This indicates that changes in the light chain ratio may not only be a direct outcome of certain diseases but also serve as an early signal of disease risk. Nevertheless, such a single ratio change may not comprehensively reflect the complex impact of diseases on the metabolism of light chains in the immune system, as found in our research results. In another study on multiple myeloma, variations in the levels of FLCs and TLCs suggested that when one type of monoclonal IgLCs (κ or λ) is overproduced, the production of the other type of light chain tends to be inhibited [24]. Therefore, we have decided to explore a more comprehensive method of assessing light chain ratio changes, specifically, a composite indicator that can reflect the relative changes in the proportions of light chains in both their free and total (bound) states.

Specifically, we sought to compare the proportions of the κ and λ types of FLC with the respective type of TLC, that is, the ratio of FLC κ/TLC κ to FLC λ/TLC λ. This method of calculation not only takes into account the total amount of IgLCs but also considers their distribution in different states, thereby enabling a more accurate reflection of the impact of disease on IgLCs metabolism. Our results indicated that this ratio, which we subsequently named the CHI, was significantly lower among CHD patients than control patients, suggesting that it may serve as a potential biomarker for CHD risk assessment. Moreover, to exclude the possibility that the differences in this index reflect underlying kidney damage [25], we additionally tested three major renal function assessment indicators in the peripheral blood of the patients, namely, creatinine, urea, and uric acid, and found no abnormalities in the CHD group. This discovery represents an important step in understanding the relationship between the immune system and cardiovascular health.

In our study, we found that the mean TLC κ/λ ratio was approximately 1.5, and the mean ratio of FLC κ/λ was approximately 1, which falls within the normal range provided by the reagent manufacturer. Therefore, a clear challenge arises: in terms of utilizing IgLCs for diagnosing diseases other than multiple myeloma and plasma cell dyscrasias, such as CHD, current approaches involving concentrations or ratios of κ and/or λ light chains cannot meet current diagnostic needs. Therefore, we postulate that the diagnostic model we devised, which incorporates the CHI, a comprehensive index that takes into account both the total amount of IgLCs and their distribution in different states, into an XGBoost-based model, has the potential to serve as a solution to this challenge. Among the nine machine learning models constructed using CHI and other clinical indicators, we identified the XGBoost-based model as the most suitable classifier for this dataset, achieving a diagnostic accuracy rate of over 85% for CHD in the final test set. Several evaluations were also conducted to further verify its generalization ability and reliability. These evaluations confirmed the stability and consistency of the model across different subsets of the data.

Notably, in this study, we found that as the severity of CHD increased (that is, as the SYNTAX score increased), the CHI gradually decreased. According to multiple pieces of evidence, coronary artery bypass grafting (CABG) is generally preferred over PCI for treating CHD when the SYNTAX score exceeds 33 or in cases involving distal bifurcation lesions or multivessel disease. However, for patients with a limited lesion range, shorter life expectancy, or high surgical risk, PCI may be a more suitable option [5, 26, 27]. The aforementioned discovery once again underscores the pivotal role of precision medicine in the treatment of CHD. Given the complex and variable conditions under which CHD can develop, a precise and personalized treatment plan is crucial, and the prompt and accurate identification of these patients is key to achieving this goal. Encouragingly, our research findings indicate that the XGBoost model has outstanding performance in the classification of patients with severe CHD according to the SYNTAX score. The AUC in the test set reached 0.991, demonstrating that the model had nearly perfect performance in identifying severe CHD patient. Moreover, the model also exhibited an accuracy of 0.857, which, alongside its desirable consistency and generalization capabilities, making it a reliable choice for CHD patient classification. Therefore, the predictive tool we have developed can serve as an auxiliary means for clinicians, providing valuable reference opinions when selecting appropriate treatment methods for patients with varying conditions of CHD.

However, it is important to acknowledge the limitations of this study, which should be considered in future research endeavors. While the CHI demonstrated promising results, but the sample size currently utilized for modeling merely meets the minimum standard, its applicability and generalizability must be further validated in larger and more diverse patient populations. This will provide a more comprehensive understanding of the index’s effectiveness across different demographic and clinical profiles. Furthermore, the current study relies heavily on the accuracy and consistency of laboratory data for IgLCs. However, it is recognized that discrepancies in detection standards and methodologies among different laboratories can potentially impact the accuracy and reproducibility of the CHI. To address this limitation, future research should focus on developing standardized detection protocols and implementing rigorous quality control measures to ensure the reliability and comparability of results across various laboratories. Lastly, regarding the online prediction tool we have developed, it is still in the testing phase. Its primary objective is to gather more external validation data in a broader context, in order to further verify the clinical utility of this tool. However, we must admit that the main limitation of this tool currently is its inability to automatically calculate CHI, which may pose certain limitations in practical applications. In our subsequent work, we will continue to optimize this tool. Despite these limitations, the findings of this study offer valuable insights into the potential of the CHI as a diagnostic or prognostic tool. As such, ongoing efforts to refine and validate the index in broader contexts will be crucial for advancing its clinical applications and ultimately improving patient outcomes.

## Conclusions

Our findings indicate that the CHI, when incorporated into an XGBoost-based model, can serve as a noninvasive, efficient, and reliable diagnostic tool that may complement existing imaging techniques and assist clinicians in making informed treatment decisions. Use of this innovative approach could lead to more precise and effective therapeutic strategies, ultimately improving patient outcomes. Future studies should aim to validate the model’s performance in larger, more diverse patient populations, explore the integration of additional biomarkers and clinical factors, and refine the model’s ability to identify subtle changes in disease progression. Additionally, research on the cost-effectiveness and clinical feasibility of implementing such a precision medicine approach in real-world settings is crucial to ensure its widespread adoption and successful implementation. Furthermore, there is a need for more longitudinal studies in the future to track and evaluate the changes in CHI over different time periods within the same patient, as well as variations across subgroups such as different age groups, genders, or those with specific complications. This will enable a more accurate determination of its long-term effectiveness in predicting the progression of CHD and assessing treatment outcomes. Such research will enhance our comprehensive understanding of the clinical significance of CHI and provide robust support for its widespread adoption and successful implementation in precision medicine.

## Electronic supplementary material

Below is the link to the electronic supplementary material.

Supplementary Material 1

## Data Availability

The study is currently ongoing. To protect the privacy of study participants, the data supporting the findings of this study are available from the corresponding author, Taiyu Zhai, upon reasonable request.

## Abbreviations

CHD: Coronary heart disease
BNP: Brain natriuretic peptide
IgLCs: Immunoglobulin light chains
TLCs: Total immunoglobulin light chains
FLCs: Free immunoglobulin light chains
CVD: Cardiovascular disease
CHI: Coronary health index
ML: Machine learning
LR: Logistic regression
RF: Random forest
AdaBoost: Adaptive boosting
GNB: Gaussian naive Bayes
MLP: Multilayer perceptron
SVM: Support vector machine
KNN: k-nearest neighbors
ROC: Receiver operating characteristic curve
AUC: Area under the curve
LCs: Learning curves
CCs: Calibration curves
DCA: Decision curve analysis
