# Supplementary material for: Coronary health index based on immunoglobulin light chains to assess coronary heart disease risk with machine learning: a diagnostic trial
Source: J Transl Med. 2025 Jan 6;23:22. doi: 10.1186/s12967-024-06043-4 (PMC11706159; doi:10.1186/s12967-024-06043-4)
Supplement: Supplementary file 1 — Supplementary Material 1 [file 12967_2024_6043_MOESM1_ESM.docx]

**Supplementary Table 1 Summary of the classification results of the different models in the training set**

| Model | AUC | Cutoff | Acc | Sen | Spec | PPV | NPV | F1 | Kappa |
| --- | --- | --- | --- | --- | --- | --- | --- | --- | --- |
| XGB | 1.0 | 0.908 | 0.996 | 0.995 | 1.0 | 1.0 | 0.987 | 0.998 | 0.991 |
| LR | 0.766 | 0.720 | 0.693 | 0.619 | 0.903 | 0.949 | 0.453 | 0.749 | 0.395 |
| LGBM | 1.0 | 0.862 | 0.996 | 0.995 | 1.0 | 1.0 | 0.987 | 0.998 | 0.991 |
| RF | 1.0 | 0.600 | 0.993 | 0.990 | 1.0 | 1.0 | 0.975 | 0.995 | 0.982 |
| Ada | 1.0 | 0.503 | 0.980 | 0.974 | 1.0 | 1.0 | 0.931 | 0.987 | 0.951 |
| GNB | 0.809 | 0.0 | 0.722 | 0.708 | 0.760 | 0.891 | 0.485 | 0.789 | 0.396 |
| MLP | 0.732 | 1.0 | 0.482 | 0.327 | 0.931 | 0.932 | 0.324 | 0.484 | 0.160 |
| DT | 1.0 | 0.680 | 0.709 | 0.664 | 0.831 | 0.913 | 0.482 | 0.768 | 0.403 |
| KNN | 0.846 | 0.800 | 0.640 | 0.511 | 1.0 | 1.0 | 0.423 | 0.676 | 0.356 |

AUC: Area under the curve. Acc: Accuracy. Sen: Sensitivity. Spec: Specificity. PPV: Positive predictive value. NPV: Negative predictive value.

**Supplementary Table 2: Summary of the classification results of the multiple models in the validation set**

| Model | AUC | Cutoff | Acc | Sen | Spec | PPV | NPV | F1 | Kappa |
| --- | --- | --- | --- | --- | --- | --- | --- | --- | --- |
| XGB | 0.920 | 0.908 | 0.852 | 0.834 | 0.940 | 0.966 | 0.657 | 0.892 | 0.658 |
| LR | 0.754 | 0.720 | 0.711 | 0.676 | 0.803 | 0.896 | 0.492 | 0.771 | 0.400 |
| LGBM | 0.918 | 0.862 | 0.831 | 0.800 | 0.938 | 0.978 | 0.585 | 0.879 | 0.608 |
| RF | 0.845 | 0.600 | 0.782 | 0.785 | 0.795 | 0.912 | 0.530 | 0.843 | 0.482 |
| Ada | 0.817 | 0.503 | 0.768 | 0.834 | 0.632 | 0.842 | 0.621 | 0.834 | 0.452 |
| GNB | 0.870 | 0.0 | 0.641 | 0.595 | 0.781 | 0.887 | 0.394 | 0.712 | 0.281 |
| MLP | 0.736 | 1.0 | 0.430 | 0.229 | 0.932 | 0.906 | 0.328 | 0.361 | 0.102 |
| DT | 0.800 | 0.680 | 0.648 | 0.649 | 0.662 | 0.868 | 0.359 | 0.740 | 0.239 |
| KNN | 0.753 | 0.800 | 0.556 | 0.439 | 0.893 | 0.924 | 0.360 | 0.591 | 0.225 |

AUC: Area under the curve. Acc: Accuracy. Sen: Sensitivity. Spec: Specificity. PPV: Positive predictive value. NPV: Negative predictive value.

**Supplementary Table 3: Comprehensive summary of all set results**

| Set | AUC | Cutoff | Acc | Sen | Spec | PPV | NPV | F1 |
| --- | --- | --- | --- | --- | --- | --- | --- | --- |
| Training | 0.965 | 0.689 | 0.858 | 0.823 | 0.974 | 0.991 | 0.629 | 0.899 |
| Validation | 0.876 | 0.689 | 0.803 | 0.796 | 0.824 | 0.940 | 0.566 | 0.859 |
| Test | 0.927 | 0.707 | 0.878 | 0.865 | 0.917 | 0.97 | 0.688 | 0.914 |

AUC: Area under the curve. Acc: Accuracy. Sen: Sensitivity. Spec: Specificity. PPV: Positive predictive value. NPV: Negative predictive value.

**Supplementary Table 4: Summary of the classification results of the multiple models in the training set**

| Model | AUC | cutoff | Acc | Sen | Spec | PPV | NPV | F1 | Kappa |
| --- | --- | --- | --- | --- | --- | --- | --- | --- | --- |
| XGB | 1.000 | 0.781 | 0.991 | 0.983 | 1.000 | 1.000 | 0.981 | 0.991 | 0.982 |
| LR | 1.000 | 0.902 | 0.991 | 0.982 | 1.000 | 1.000 | 0.982 | 0.991 | 0.982 |
| LGBM | 1.000 | 0.625 | 0.986 | 0.974 | 1.000 | 1.000 | 0.973 | 0.987 | 0.973 |
| RF | 1.000 | 0.580 | 0.991 | 0.983 | 1.000 | 1.000 | 0.981 | 0.991 | 0.982 |
| Ada | 0.891 | 0.000 | 0.842 | 0.882 | 0.804 | 0.817 | 0.877 | 0.847 | 0.685 |
| GNB | 0.612 | 1.000 | 0.450 | 0.000 | 1.000 | NA | 0.450 | NA | 0.000 |
| MLP | 0.813 | 0.490 | 0.757 | 0.808 | 0.696 | 0.758 | 0.756 | 0.782 | 0.507 |
| SVM | 0.859 | 0.700 | 0.631 | 0.322 | 0.991 | 0.981 | 0.561 | 0.473 | 0.299 |
| KNN | 0.796 | 0.515 | 0.757 | 0.686 | 0.834 | 0.827 | 0.700 | 0.748 | 0.514 |

AUC: Area under the curve. Acc: Accuracy. Sen: Sensitivity. Spec: Specificity. PPV: Positive predictive value. NPV: Negative Predictive Value. 237 samples were included in this analysis.

**Supplementary Table 5: Summary of the classification results of the multiple models in the validation set**

| Model | AUC | cutoff | Acc | Sen | Spec | PPV | NPV | F1 | Kappa |
| --- | --- | --- | --- | --- | --- | --- | --- | --- | --- |
| XGB | 0.977 | 0.781 | 0.946 | 0.931 | 0.964 | 0.964 | 0.929 | 0.947 | 0.893 |
| LR | 0.955 | 0.902 | 0.839 | 0.750 | 1.000 | 1.000 | 0.683 | 0.857 | 0.676 |
| LGBM | 0.972 | 0.625 | 0.929 | 0.867 | 1.000 | 1.000 | 0.871 | 0.927 | 0.858 |
| RF | 0.977 | 0.580 | 0.857 | 0.763 | 0.958 | 0.964 | 0.782 | 0.850 | 0.714 |
| Ada | 0.803 | 0.000 | 0.714 | 0.639 | 0.850 | 0.887 | 0.567 | 0.742 | 0.440 |
| GNB | 0.581 | 1.000 | 0.57 | 0.000 | 1.000 | NaN | 0.571 | NaN | 0.000 |
| MLP | 0.667 | 0.490 | 0.643 | 0.731 | 0.567 | 0.598 | 0.703 | 0.657 | 0.294 |
| SVM | 0.732 | 0.700 | 0.518 | 0.033 | 0.962 | NaN | 0.516 | NaN | 0.005 |
| KNN | 0.779 | 0.515 | 0.679 | 0.644 | 0.760 | 0.705 | 0.687 | 0.649 | 0.370 |

AUC: Area under the curve. Acc: Accuracy. Sen: Sensitivity. Spec: Specificity. PPV: Positive predictive value. NPV: Negative predictive value. 237 samples were included in this analysis.

**Supplementary Table 6: Prediction accuracy of online tool**

| Set | Total | Correct | Accuracy rate (%) |
| --- | --- | --- | --- |
| CHD | 59 | 51 | 86.44 |
| Severe lesions | 12 | 12 | 100 |

**Supplementary Figure 1**


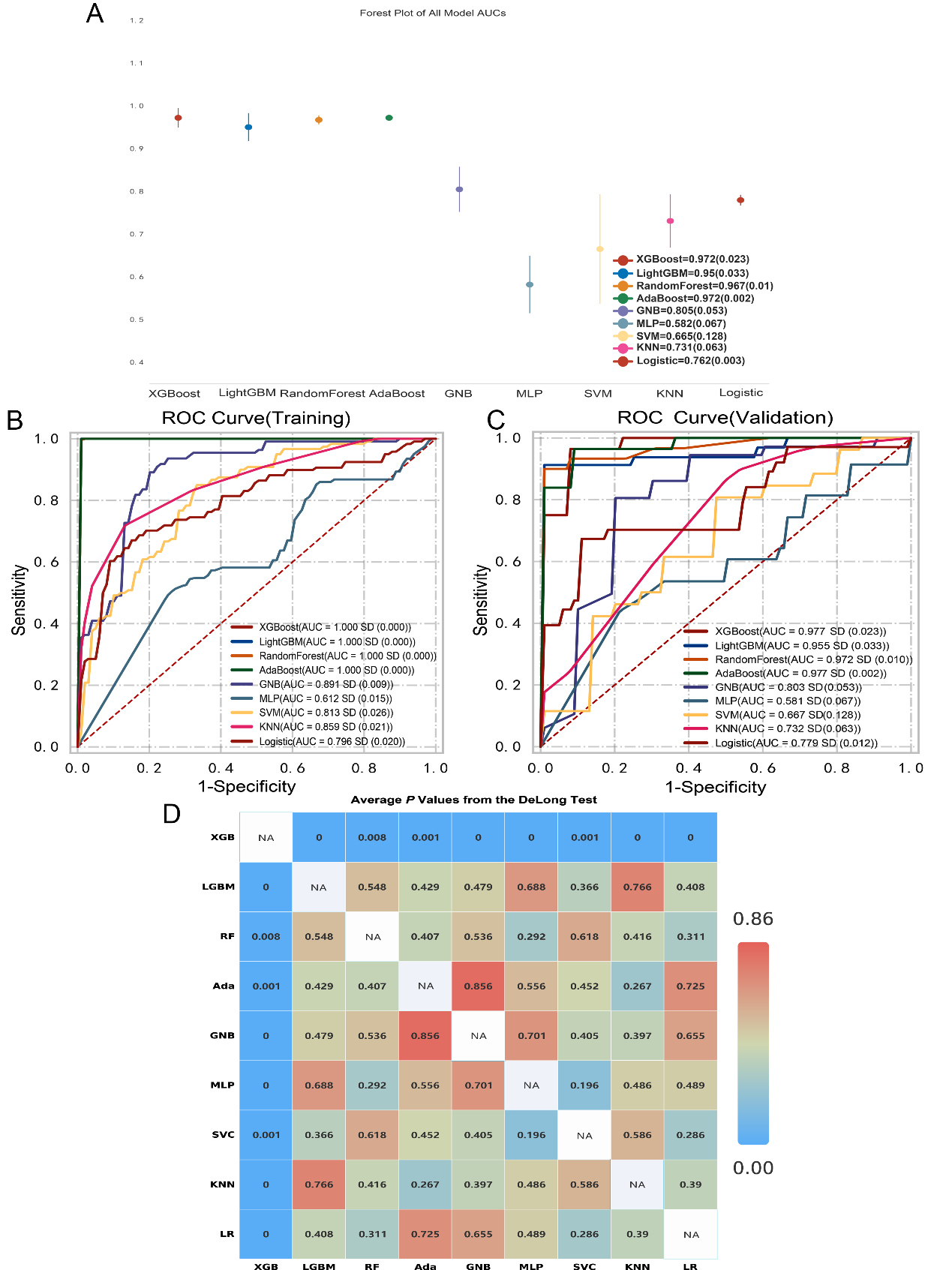


**Supplementary Figure 1: XGBoost is the optimal ML model for distinguishing controls from severe CHD patients.**

(A) The forest plot displays the AUCs of the CHD prediction models, with error bars indicating the means and SDs. Through 2-fold cross-validation, XGBoost emerged as the top performer in the training set, with the AUC. (B) The AUC value of the XGBoost model in the training set was the highest. (C) The XGBoost classification model performed the best in the validation set. (D) The DeLong test results indicate that the AUC of the XGBoost model is significantly different from the AUCs of the other models.
